# Khat (*Catha Edulis*) as a Risk Factor for Cardiovascular Disorders: Systematic Review and Meta-Analysis

**DOI:** 10.2174/1874192401711010146

**Published:** 2017-12-19

**Authors:** Teshale Ayele Mega, Nikodimos Eshetu Dabe

**Affiliations:** 1 School of Pharmacy, Institute of Health Science, Jimma University, Jimma, Ethiopia; 2 Department of Biomedical Science, College of Health Science, Mizan Tepi University, Mizan Teferi, Ethiopia

**Keywords:** Khat, Cardiovascular system, Meta-analysis, Systematic Review, Blood pressure, Heart rate

## Abstract

**Background:**

About 20 million people worldwide are believed to be using khat. Although some studies reported that khat chewing might result in cardiovascular disorders, conclusive evidence is limited.

**Method:**

The objective of this review was to synthesize the best available evidence for the effect of khat on the cardiovascular system. Databases searched were PubMed, Cochrane database of systematic reviews, CINAHL, poplin, LILACS, MedNar and Scopus. All papers included in the review were subjected to rigorous appraisal using the Joanna Briggs Institute (JBI) standardized critical appraisal tool. Review Manager Software (Revman 5.3) was used for meta-analysis and effect size and the 95% confidence interval (CI) was calculated.

**Result:**

Data was extracted from 10 articles. Our meta-analysis included 9,207 subjects, (2123 chewers and 7084 non-chewers, respectively) to elucidate the effect of khat on heart rate, diastolic and systolic blood pressure. The mean diastolic and systolic blood pressure of khat chewers was higher than the non-chewers with a mean difference of 5.1 mmHg, 95%CI [2.7,7.5] and 7.9 mmHg, 95%CI [2.65, 13.18], respectively. Similarly, the heart rate of the chewers remained consistently higher, making the mean difference of 6.9 beats/min, 95%CI [0.5, 13.3]. In addition, khat was found to have either a causative or worsening effect on stroke, myocardial infarction and heart failure.

**Conclusion:**

We showed that khat chewing could significantly affect the cardiovascular system through its effect on heart rate and blood pressure. Therefore, health promotion should be aimed to encourage quitting khat chewing.

## 1 INTRODUCTION

The khat plant was first described during an expedition to Egypt and Yemen in 1761-1763 by a Swedish botanist named Peter Forskal, who identified *Catha edulis* as a member of the family *Celastraceae* [1]. Khat (also known as Qat, Kat and Miraa) is a dicotyledonous evergreen flowering tree that grows in the equatorial climates mainly in the Arabian Peninsula and the regions around the horn of Africa [2]. Ethiopia, Yemen, Kenya, Madagascar and Somalia are the 5 main khat growing countries. The plant also grows to a lesser extent in Uganda, Tanzania, Rwanda, Zimbabwe, Zaire, Angola, Malawi, Mozambique, Zambia, Swaziland and South Africa [3].

About 20 million people worldwide are believed to be using khat, which previously was confined to East Africa and the Arabian Peninsula [4]. It was initially thought to be of limited concern to western populations but, overnight delivery systems and immigration of khat chewers contributed to its globalization [5]. Khat chewing is as high as 15% in Ethiopia and 90% in Yemen [6].

The leaf is the commonest part of the plant which is chewed slowly over several hours and the juice of the masticated leaves is swallowed [7]. Most people chew khat leaves for several hours per day to enjoy the taste and to experience the stimulating effect on the central nervous system (CNS) [8]. The fresh leaves of khat contains over 40 compounds of which the amphetamine like chemicals cathine and cathinone, are reported to be responsible for its CNS and cardiovascular system (CVS) effects [9]. The effects of these compounds on the CVS are expressed by an increment in heart rate (HR), blood pressure (BP), and vasomotor effects on the coronary vessels [10]. In one study, the administration of cathinone produced clear cut increases in BP and HR [11] and regular khat use was shown to cause a rise in BP and HR. These changes may parallel the levels of cathinone in the plasma [12]. A study conducted in Yemen found that the rise in BP corresponded to the duration of khat chewing [13]. However, a recently conducted review [14] failed to produce an evidence for khat to be a risk factor for development of hypertension, major risk factor for other cardiovascular complications.

In addition to its effects on BP, khat has also been associated with the increased incidence of acute coronary vasospasm and myocardial infarction (MI) [15]. As one of the constituents of khat, cathinone is reported to be associated with severe coronary vasoconstriction and a severe negative inotropic effect on the cardiac muscle, suggesting that coronary spasm contributes to the development of acute MI [10, 16]. Furthermore, Al-Shami and Al-Motarreb, evaluated the effect of khat chewing on the coronary arteries in patients with history of heart failure. They found that a history of chewing khat was an independent risk factor for coronary heart disease (CHD) [17]. Other researchers have also reported that both the intensity (quantity) and duration of khat use could contribute to the development of MI [18-20].

Khat-associated acute coronary syndrome (ACS) will also lead to worse outcomes [4]. Khat chewers also had a higher risk of death, recurrent myocardial ischemia, cardiogenic shock, and ventricular arrhythmia [4]. Khat chewing was also found to be an independent risk factor of death, recurrent cardiac ischemia, heart failure and stroke [3]. There were also several case reports from different parts of the world showing that, khat chewing was commonly associated with severe ischemic cardiomyopathy and stroke [21-23].

The medical and socioeconomic problems related to the use of khat have attracted the attention of international organization [24]. Through the United Nations Commission on Narcotic Drugs, international attention was directed to the nature and extent of khat use and in 1971 the Commission recommended that, the United Nations Narcotics Laboratory should reinvestigate the chemical composition of khat [25]. the level of abuse and threat to public health is not significant enough to warrant international control [26]. Nevertheless, some countries including Finland, Germany, New Zealand, Sweden, France, Norway, Denmark, Canada, United State of America (USA), United Kingdom (UK) and Saudi Arabia [27], have prohibited khat consumption.

Overall, the current understanding and evidence on the health effects of khat are inconclusive despite the ever-growing rate of use behaviors [28]. Therefore, we evaluated the evidence concerning cardiovascular risk and khat use.

## 2 METHODS

The objective of this review was to systematically identify, appraise and synthesize the best available evidence for the effect of khat on the CVS. All relevant and available peer-reviewed human studies published in English until May 2017 were considered, regardless of age, sex, race, country of residence, khat dose, frequency, duration of chewing or other characteristics of the chewers. Articles were excluded from the meta-analysis if they compared the combined effect of khat and other substances.

A three staged search strategy was used to identify all relevant published literature in English language. Databases searched were PubMed, CINAHL, popLine, LILACS, MedNar and Scopus. Secondary search was carried out from Google Scholar in identifying articles that are not indexed well in traditional bibliographic databases. The following search strategy or its modified form with initial keywords/search terms was used for various databases and search engines: [“*Catha edulis*” or “Khat” or “Mairungi” or “Miraa” or “Chat” or “Qat plant”] and [“Cardiovascular” or “Cardiovascular System” or “Circulatory System” or “Heart” or “Blood Vessels” or “Blood Pressure” or “ Heart Rate” or “Heart failure” or “Stroke”]. The first search was conducted from November 10-28/2016 and the search was updated on 23 May 2017. All papers of optimal quality were selected for inclusion and those articles without optimal data set for meta-analysis were subjected to narrative synthesis. Articles selected for critical appraisal were assessed by 2 independent reviewers for methodological validity using standardized critical appraisal instruments from the Joanna Briggs Institute Meta-Analysis of Statistics Assessment and Review Instrument (JBI-MAStARI) https://www.joannabriggs.org/assets/docs/jbc/...sr.../jbi-sr-protocol-template.docx. Any disagreements between the reviewers were resolved by discussion.

### 2.1 Primary Study Identification and Data Extraction

We extracted the evidence from original articles, which were assessed by at least one of the following outcomes: hypertension (raised systolic or diastolic BP), and tachycardia or increased HR were considered as the primary outcomes. For observational studies, before BP measurement, each participant was advised to rest for at least 5 min. While for randomized clinical trials, BP and HR were measured at zero time (30 min before khat chewing) and at 1 h, 2 hand 3 h during khat chewing as well as 1 h after spitting out the leaves.

The BP was measured using a mercury based cuff sphygmomanometer on the bared arm in the sitting position 3 times at 5 min intervals. The mean reading was taken. MI, stroke, and cardiomyopathy were considered as secondary outcome. We extracted outcome using the standardized data extraction tool of JBI-MAStARI (https://www.joannabriggs.org/assets/docs/jbc/...sr.../jbi-sr-protocol-template.docx). All outcomes were extracted by 2 independent reviewers to avoid errors.

### 2.2 Data Analysis

Review manager version 5.3 was used for data analysis and a random-effect meta-analysis was conducted to pool the mean for each of the outcomes. Forest plots including mean, standard deviation and confidence intervals (CI), p value, effect size, and, heterogeneity (I^2^) were constructed. Mean differences with their p values <0·05 were considered significant.

## 3 RESULTS

**Fig. (1):**
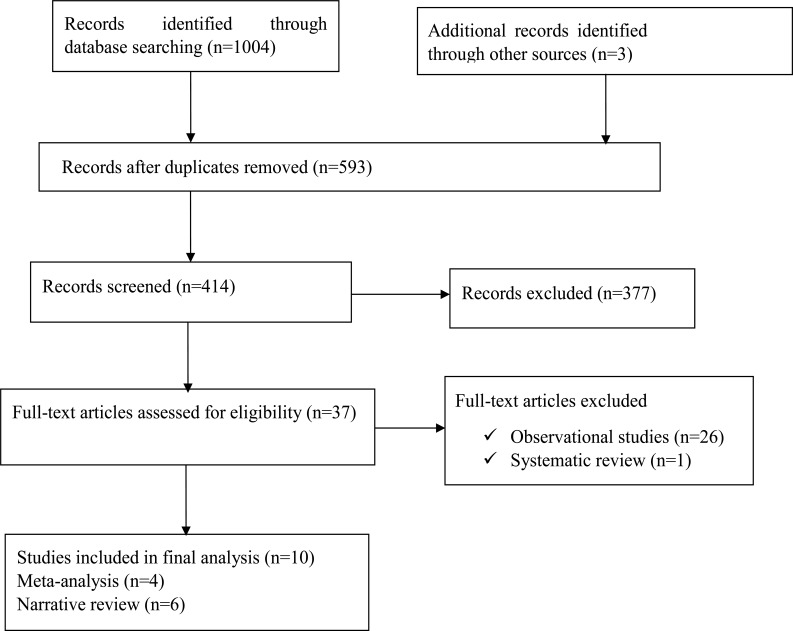
Flowchart of study selection for inclusion in to the evidence synthesis for the effect of khat on the cardiovascular system.

Over all, there were 1004 records identified through searching from the mentioned databases; 37 full articles were identified for eligibility and 10 of them were included in the final review (Fig. **1**).

**Fig. (2):**
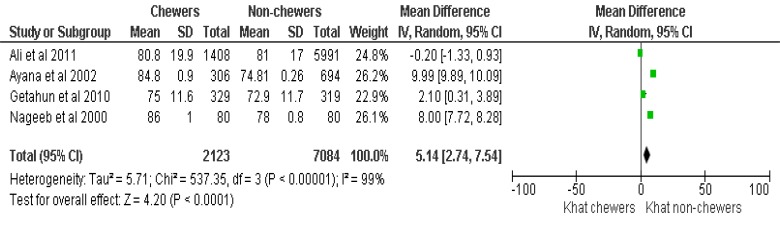
The effect of Khat on the diastolic blood pressure.

**Fig. (3):**
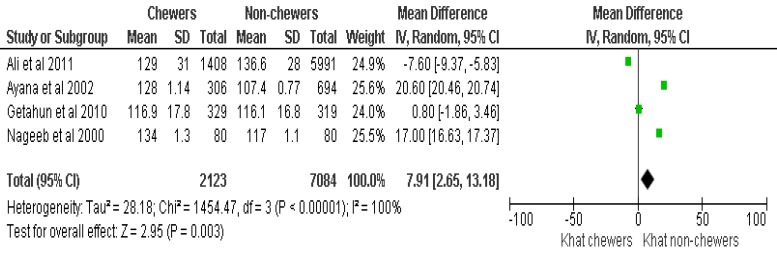
The effect of Khat chewing on systolic blood pressure.

**Fig. (4):**
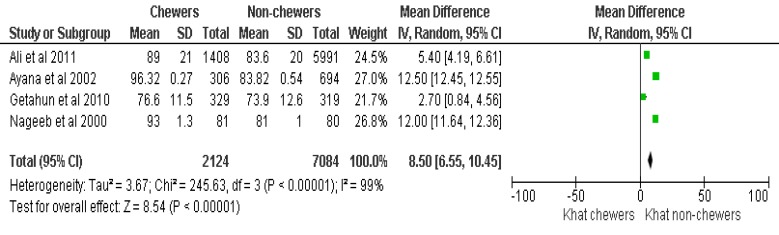
Effect of khat chewing on heart rate.

Of the 10 eligible articles, 4 articles (1 Randomized Controlled Trial and 3 observational studies) (Table **1**) were included in the final meta-analysis and 6 articles were used for narrative review (Table **2**) to demonstrate the effect of khat on diastolic BP (DBP), systolic BP (SBP) and HR (Figs. **2**, **3** and **4**).

Overall data from 9207 subjects, (2123 chewers and 7084 non-chewers, respectively) with a chewers to non-chewers ratio of 1:3.3 was included to synthesize the evidence for the effect of khat on DBP. The meta-analysis results showed that, the Mean DBP of khat chewers was higher with the mean difference of 5.1 mmHg, 95% CI [2.7,7.5]. Before BP measurement, each participant was advised to sit and take rest for at least 5 min. Three consecutive measurements were taken on the left arm at 3-5 min intervals. The average of the second and third measurements was used to describe the mean SBP and mean DBP and HR. The overall effect was statistically significant (p<0.0001) and the summary effect of the meta-analysis was: Heterogeneity: Tau^2^= 5.72, Chi^2^=536.50, df =3(p<0.00001), I^2^=99% (Fig. **2**).

The analysis for synthesizing evidence regarding SBP also considered a similar dataset as used for DBP. Accordingly, khat chewers had higher mean SBP, with the mean difference of 7.9 mmHg, 95%CI [2.6, 13]. The test for the overall effect was; Z=2.9 (p=0.003). The summary effect of the meta-analysis was: Heterogeneity: Tau^2^=28.18; Chi^2^=1454.47, df =3 (p<0.00001); I^2^=100% (Fig. **3**).

To demonstrate the effect of khat on HR, the data of 9,207 subjects, with a chewer to non-chewer ratio of 1:3.3, was considered. The HR of the chewers was found to be consistently elevated with a mean difference of 6.9 beats/min, 95% CI [0.5, 13.3]. Despite heterogeneity reported under the summary effect of meta-analysis, the overall effect of the mean difference (Z=8.5 (p<0.00001) in HR remained significant, (Heterogeneity: Tau^2^=31.7; Chi^2^=238.84; df =2(p<0.00001); I^2^=99%), (p=0.03) (Fig. **4**).

The observed heterogeneity, as shown above in the meta-analysis results, might not influence the finding as the reviewers passed their critical appraisal and all the outcomes have uniform direction of effect measure. The statistical heterogeneity could be attributed to clinical heterogeneity of the study subjects as the analysis did not consider prior clinical characteristics of the subjects or the dose and duration of khat consumed.

There are also reports from other preliminary studies that supplement the above findings. Birhane *et al.,* 2014 [29] reported that, out of the total khat chewers, the majority (85.3% and 67.1%) of the participant’s SBP and DBP was >120 and 80 mmHg, respectively. Another study showed that, the prevalence of hypertension (SBP ≥140 mmHg or DBP ≥90 mmHg) or reported use of antihypertensive drugs was significantly higher among khat chewers (13.4%) than non-chewers (10.7%), with the adjusted odds ratio of (AOR = 1.66; 95% CI [1.05, 3.13] [30]. Fikru *et al.*2008 [31] also reported that, regular khat chewing was significantly associated with elevated mean DPB (β = 1.9, p = 0.02). There were also similar findings from Yemen [28]. In addition to elevation of BP and HR, khat chewers were at higher risk of developing AMI and stroke [4, 10, 16-19, 22]. We did not include some of the above studies in to the meta-analysis because they did not quantify the intended outcome.

## 4 DISCUSSION

The shift in the global burden of disease from communicable, maternal, perinatal and nutritional causes to non-communicable diseases (NCDs) [32] has become a major challenge. By 2020, heart disease and stroke will become the leading causes of global death and disability [33]. The projected number of fatalities is expected to rise to >24 million by 2030, with > 80% of the deaths occurring in low and middle income countries [33]. The estimated percentage of premature deaths from CVDs ranges from 4% in high-income countries to 42% in low-income countries, leading to growing inequalities [34].

Conventional risk factors for CVS have been identified and interventions have made considerable progress [35]. However, studies on specific substances like khat chewing were not conducted well or interventions were not carried out. Considering the growing prevalence of khat chewing in Africa and worldwide [9, 30, 36], the findings of this review should alarm the organizations working on public health issues. The implications are important as abnormal increases in BP and HR are key risk factors for the pathogenesis of CVD [37, 38].

Few attempts were made to assess the cause and effect relationships of khat with CVD. A Study conducted in Yemen reported that the increase in BP and HR in khat-chewers coincided with raised plasma cathinone concentrations [19]. Accordingly, about 59% of khat chewers had onset of symptoms of AMI during the khat-effective period and only 36.4% of non-khat chewers had a new onset of AMI symptoms [19]. A review showed that regular khat chewing was associated with elevated mean DBP [8].

The present findings were also consistent with a review [10], which assessed the effect of khat on heart failure. The authors of that review proposed that khat could significantly affects CVS by increasing catecholamine release, HR, BP, and inducing coronary vasospasm. A finding by Ahmed *et al*. [39] also supplements the results of the present review since persistent elevation in BP and HR was observed among khat chewers.

Studies on human subjects with primary outcome of assessing effect of khat on HR and BP are too few or date back decades considering the very high custom of khat chewing habits across residents of East Africa and the Arabian Peninsula [29]. However, most of the available studies describe the negative effect of khat on cardiovascular outcome [4, 29-31, 40].

In a study by Motarreb *et al*. [18], mild chewers were not shown to be at risk of AMI, while moderate khat chewers were shown to be at high risk (OR = 7.62) and heavy khat chewers at even higher risk (OR = 22.28). These findings study are in line with animal studies intended to establish cause and effect relationship. In an animal study, a marked constriction of the coronary vasculature, the maximum being equivalent to that achieved with noradrenaline or the cathinone metabolite, nor- pseudoephedrine [29]. The pronounced negative inotropic effect, possibly due to the impaired coronary perfusion was demonstrated in isolated perfused hearts of guinea-pigs [29, 41].

Appropriate care of patients with cardiovascular conditions like hypertension, heart failure, ACS including their diet, physical activity, medical care, together with early detection, and complications management can significantly reduce disability and early mortality [42]. However, such interventions are costly compared with primary prevention options as most complications associated with cardiovascular problems need a more advance care.

One of the strengths of the present study is the consideration of multiple outcomes and inclusion of more literature as compared with the previous reviews conducted to establish the impact of khat on BP [14.] This enabled the authors to provide that khat is a risk factor for elevated BP, and tachycardia, which in turn are major risks for other cardiovascular disorders. Thus, we were able to overcome the limitation of the review by Kalkidan et al [14], which concluded that there was insufficient evidence that khat was a risk factor for hypertension. The findings of our study are limited by inclusion of few articles, and poor methodological quality data, as we only included 1 RCT [43], which scored only 1point on the Jadad scale for reporting RCTs [44]. The issue of heterogeneity was another limitation of this review. In addition, collecting data from different designs, ignorance of the khat chewing duration and the quantity of khat consumed were another issues to be considered.

Therefore, we urge for cautious interpretation of the study findings and there is also a need for further review involving studies with high quality design, to assess the cardiovascular implications of khat use.

## CONCLUSION AND RECOMMENDATIONS

This systematic review showed that, khat chewing in different countries is associated with high BP and elevated HR, which are established risk factors for cardiovascular diseases. Considering, the impact of this plant on the economy, the governments of these regions should design appropriate strategies like, imposing heavy taxation on khat trade, improving youth recreational services and creating adequate job opportunities. As the poor or jobless are a group mostly engaged in khat chewing practice, increasing the price of khat could be solution.

Health professionals should also play a role in promoting the health impacts of khat and provide psychosocial support services to quit the khat chewing habit for those who are affected chronically. The global community should also work together to reduce or halt the rate of border crossing khat trade. Generally, clear policies should be designed and implemented to curb khat chewing in those countries with the most at risk populations.

## Figures and Tables

**Table 1:** Description of the studies included in the meta-analysis.

**Author**			**Study design**	**Outcome assessed**	**Country**
	**Chewers**	**Non- chewers**			
Ali *et al.,* 2011 [4]	1406	5993	Cohort	Chewers were more likely to develop hypertension, tachycardia and die compared with non-chewers (7.5 vs. 3.8%; p ˂ 0.001). Chewing was also associated with heart failure, recurrent MI, ventricular arrhythmia and cardiogenic shock.	Middle Eastern Gulf countries
Getahun *et al.,* 2010 [30]	334	330	Cross-sectional	Prevalence of hypertension (SBP >140 mmHg or DBP >90 mmHg was significantly higher among chewers (13.4%) than non-chewers (10.7%), OR = 1.66 (95% CI 1.05, 3.13).	Ethiopia
Ayana *et al*., 2002 [41]	306	694	Cross-sectional	About 22.88% of khat chewers were hypertensive (p < 0.001), a higher value than that of non-chewers. Significant association between khat chewing and tachycardia (p < 0.001).	Ethiopia
Nageeb *et al.,* 2000 [43]	80	80	RCT	Significant and progressive elevation of all mean BP parameters and HR at hourly intervals after starting to chew khat compared with baseline values.	Yemen

**BP**: blood pressure; **DBP:** diastolic blood pressure; **HR**: heart rate; **MI**: myocardial infarction; **OR**: odds ratio; **RCT**: randomized control trial; **SBP**: systolic blood pressure

**Table 2:** Description of studies included in the narrative review.

**Author**			**Design**	**Outcome assessed**	**Country**
	**Khat Chewers**	**Non- chewers**			
Al-Motarreb *et al.,* 2005 [18]	124	33	Case control	Increases in BP and HR observed in chewers which coincide with raised plasma cathinone concentrations and 59% of khat chewers had onset of symptoms of AMI during the khat-effective period, compared with only 36.4% of non-chewers.	
Alkadi *et al*.,2002 [19]	95	25	Case control	The occurrence of MI after chewing is more than that before chewing and is more than that of during chewing. Chewing may be considered as a risk factor for occurrence of MI especially in persons who are susceptible to the disease.	Yemen
akajima *et al.,*2014 [27]	49	52	Cross-sectional	Significant effects of khat only group were found in SBP (F [2,139] = 8.48, p < 0.001) and DBP (F [2,135] = 4.69, p =0 .01). Also, significant effects were found in both SBP (p <0.002), and DBP (p< 0.01) in khat and tobacco users relative to non-users.	Yemen
Birhane *et al.*, 2014 [29]	422	-	Cross-sectional	Majority of chewers, 85.3% and 67.1% of the participants had a SBP and DBP >120 and 80 mmHg respectively.	Ethiopia
Tesfaye *et al.,* 2008 [31]	636	3365	Cross-sectional	Regular khat chewing was associated with elevated mean DBP (β = 1.9, p = 0.02). Khat chewing among men was associated with high BP, an established risk factor for CVD	Ethiopia

## LIST OF ABBREVIATIONS

AMI: acute myocardial infarction
AOR: adjusted odds ratio
CVD: cardiovascular disorders
CVS: cardiovascular system
DBP: diastolic blood pressure
HR: heart rate
MI: myocardial infarction
NCDs: non communicable diseases
OR: odds ratio
SBP: systolic blood pressure
UK: United Kingdom
USA: United States of America

## AVAILABILITY OF DATA AND MATERIALS

Data sharing not applicable to this article as no datasets were generated or analysed during the current study
